# Complement Factor H Gene Variant in a Patient with Thrombotic Microangiopathy on a Mixed Clinical Background

**DOI:** 10.1155/2021/2519918

**Published:** 2021-10-25

**Authors:** Yoichi Iwafuchi, Tetsuo Morioka, Yuko Oyama, Shin Goto, Ichiei Narita

**Affiliations:** ^1^Department of Internal Medicine, Koseiren Sanjo General Hospital, 5-1-62 Tsukanome, Sanjo 955-0055, Japan; ^2^Department of Internal Medicine, Kidney Center, Shinrakuen Hospital, 3-3-11 Shindoriminami Nishi-ku, Niigata 950-2087, Japan; ^3^Division of Clinical Nephrology and Rheumatology, Niigata University Graduate School of Medical and Dental Sciences, 1-754 Asahinachi-Dori, Niigata 951-8510, Japan

## Abstract

We report the case of a patient with *complement factor H* gene variant, who developed thrombotic microangiopathy on a mixed clinical background. A 79-year-old woman was transferred to Sanjo General Hospital for maintenance hemodialysis. She suffered from gastric non-Hodgkin lymphoma about two years ago and received chemotherapy and radiation therapy, leading to complete remission. About 13 weeks prior to her transfer to our hospital, she was referred to another hospital due to acute kidney injury, hemolytic anemia, and thrombocytopenia. Hemodialysis was immediately initiated, after which intravenous methylprednisolone and oral prednisolone were started; however, she became anuric within approximately week. The possibility of thrombotic microangiopathy was examined. However, she was in poor general condition and did not get the consent of her family, so no invasive searches such as a kidney biopsy were performed. Despite the cause of acute kidney insufficiency being unclear, she was transferred to us for maintenance hemodialysis. Her general condition was stable, and her renal function improved; hence, two months after transfer, a kidney biopsy was performed. Her clinical and typical renal histological findings indicated a diagnosis of thrombotic microangiopathy. There was a possible CFH gene of a very rare variant “c.526 T > *C* (p.Phe176Leu)” in exon 5. She was able to withdraw from hemodialysis therapy two weeks after the initiation of an angiotensin-converting enzyme inhibitor. Based on her clinical course and kidney biopsy findings, she was diagnosed with thrombotic microangiopathy with a very rare CFH variant. To ensure proper treatment choices such as eculizumab, the presence of complement dysregulation should be considered in cases of secondary thrombotic microangiopathy.

## 1. Introduction

Thrombotic microangiopathy (TMA) represents a heterogeneous group of syndromes with the same phenotype: a clinical triad of microangiopathic hemolytic anemia, thrombocytopenia, and organ damage [1]. The pathophysiology of TMA is not fully understood. However, it is likely a multifactorial disease in which various genetic and environmental factors overlap. Accurate diagnosis is important because the causes of TMA are diverse, and the treatment and prognosis differ depending on the cause.

The classification of TMA is currently controversial. TMA can be divided into primary (genetic and acquired) and secondary causes [1]. The concept of atypical hemolytic uremic syndrome (aHUS), a type of TMA that has been clarified in recent years, is that it is caused by complement overactivation in the alternative pathway [2]. aHUS, referred to as complement-mediated TMA, is a syndrome that includes congenital caused by multiple complement genes and various acquired causes [2]. aHUS develops when environmental factors are added to genetic factors, but not all can be explained by genetic factors. There are various pathological conditions that cause secondary TMA. In recent years, attention has also been focused on complement gene abnormalities in secondary TMA because these are related to treatment choices, specially to the use of eculizumab (ECZ) [3].

Here, we report the case of a patient with *complement factor H *(CFH) gene variant, who developed TMA on a mixed clinical background, and discuss the possibility of complement dysregulation being present in secondary TMA.

## 2. Case Presentation

**Figure 1:**
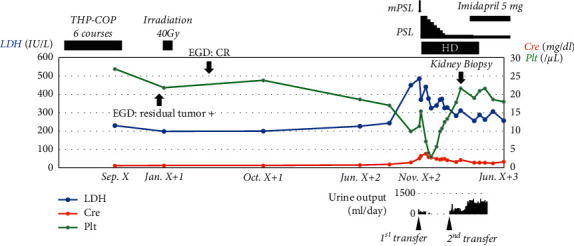
Clinical course of the patient. THP-COP: combination of pirarubicin, cyclophosphamide, vincristine, and prednisolone, EGD: esophagogastroduodenoscopy, CR: complete response, mPSL: methyl prednisolone pulse therapy, PSL: prednisolone, HD: hemodialysis, LDH: lactate dehydrogenase, Cre: creatinine, and Plt: platelet.

A 79-year-old woman was transferred to Sanjo General Hospital for maintenance hemodialysis and rehabilitation. She had no family history of renal disease. Her medical history was complex. About two years before transfer, she was diagnosed with gastric non-Hodgkin lymphoma (diffuse large B-cell type) in a medical examination at the first hospital. She was treated with six cycles of rituximab, tetrahydropyranyl adriamycin, cyclophosphamide, vincristine, and prednisolone. These drugs, excluding prednisolone, were not used since then. However, two months after her last chemotherapy for lymphoma, a residual tumor was confirmed. Definitive radiotherapy was performed with a curative intent, totaling 40 Gray in 20 fractions. A complete response was confirmed by an upper gastrointestinal endoscopy after radiotherapy, with no progression observed. After 17 months, about 13 weeks prior to her transfer to our hospital, she was noted to have proteinuria, renal dysfunction, hemolytic anemia, and thrombocytopenia for the first time on follow-up medical examinations. She was referred to the second hospital because of acute kidney injury (AKI) with hemolytic anemia and thrombocytopenia (platelet count was 99,000/*µ*L). Her physical examination findings on to the second hospital were as follows: height, 158.0 cm; weight, 53.0 kg; blood pressure, 136/88 mmHg; and body temperature, 36.8°C. On admission, urinalysis revealed microscopic hematuria (urinary sediment, 50–99 erythrocytes/high-power field) and the urine protein/creatinine ratio was 4.7. Her hematocrit was 30.5%, hemoglobin concentration was 9.9 g/dL, platelet count was 178,000/*µ*L, and leukocyte count was 6,690/*µ*L. Red cell fragments were observed on peripheral blood smear. Her serum urea nitrogen level was 36 mg/dL, creatinine (Cre) level was 2.55 mg/dL, and lactate dehydrogenase level was 485 IU/L. The total complement level was 14 (reference range: 30–46) IU/L, complement 3 (C3) level was 41.8 (73–138) mg/dL, and complement 4 (C4) level was <5 (11–31) mg/dL. Additional laboratory data at the second hospital are summarized in Table 1. Abdominal ultrasound revealed mildly atrophic kidneys. Her renal function very rapidly worsened, and she had a severely high blood pressure (200/100 mmHg). Assuming malignant hypertension and a collagen vascular disease-related kidney injury such as lupus nephritis, hemodialysis was immediately initiated; simultaneously, a 3-day course of intravenous methylprednisolone (500 mg/day) followed by oral prednisolone at 45 mg/day was started; however, she became anuric in about a week. The possibility of TMA was investigated. However, she was in poor general condition and did not get the consent of her family, so no invasive searches, such as a kidney biopsy, were performed. Prednisolone was gradually tapered to a dose of 5 mg/day (Figure 1). She was transferred to Sanjo General Hospital for rehabilitation and maintenance hemodialysis.

When we carefully reassessed her, we observed that her urine volume had increased to 700 mL/day. In addition, since her serum Cre level before dialysis was very low (1.59 mg/dL), steroid treatment seemed to be effective to some extent. Kidney atrophy was unnoticeable, so with the consent of her and her family, we performed kidney biopsy to investigate the cause of renal failure.

**Figure 2:**
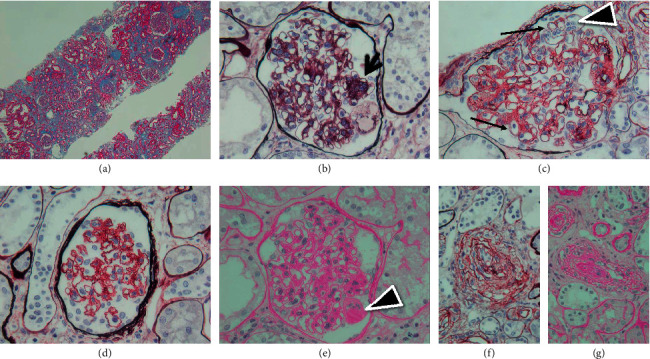
(a) Light microscopy of the biopsied sample (Masson's trichrome stain, original magnification ×48). (b) Segmental endocapillary hypercellularity (arrow) (periodic acid-silver methenamine stain, original magnification ×400). (c) Focal double contours without spike formation (arrows) and mesangiolysis (arrowhead) (periodic acid-silver methenamine stain, original magnification ×400). (d) Wrinkling of the glomerular basement membrane (periodic acid-silver methenamine stain, original magnification ×400). (e) Segmental thrombus in one glomerulus (arrowhead) (periodic acid-Schiff stain, original magnification ×400). Some arterioles revealed thickness of the arterial intima and narrowing or complete obstruction of the vessel lumina. (f) Periodic acid-silver methenamine stain, original magnification ×400. (g) Periodic acid-Schiff stain, original magnification ×400.

**Figure 3:**
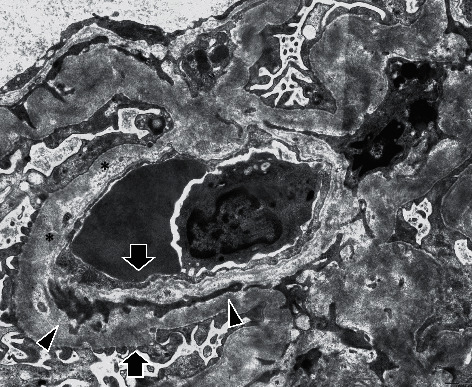
Electron microscopy revealed wrinkling of the glomerular basement membrane, expansion of the subendothelial space (asterisks), mesangial interposition (arrowhead), and reduplication of the glomerular basement membrane (arrow) (electron microscopy, original magnification ×3000).

On performing light microscopy, we observed the following: the biopsied sample contained 43 glomeruli, of which 8 showed global sclerosis, 1 showed segmental sclerosis, and 1 showed a thrombus (Figure 2(a)). Diffuse global or segmental mesangial expansion was seen, which was accompanied by mesangiolysis. Segmental endocapillary hypercellularity, focal double contours without spike formation, and mesangiolysis were observed (Figures 2(b) and 2(c)). Wrinkling of the glomerular basement membrane (GBM) was also observed (Figure 2(d)). A segmental thrombus was observed in one glomerulus (Figure 2(e)). Tubular atrophy and interstitial fibrosis were noticed (Figure 2(a)). Some arterioles revealed thickness of the arterial intima and narrowing or complete obstruction of the vessel lumina (Figures 2(f) and 2(g)). Immunofluorescent examination revealed no significant deposits of immunoglobulins or complement components. Electron microscopy revealed wrinkling of the glomerular basement membrane (GBM), expansion of the subendothelial space, mesangial interposition, and reduplication of the GBM (Figure 3).

Based on her clinical and histological findings, such as double contours and wrinkling of the GBM, mesangiolysis, and thickness of the arterial intima, she was diagnosed with TMA. The course of her disease was variable; hence, we reviewed her past medical history in detail. Gastric non-Hodgkin lymphoma had been in complete remission at the onset of TMA. She presented with severe hypertension; however, she did not meet the criteria for malignant hypertension. Although the disease was pathologically consistent with radiation nephropathy, there was no reliable evidence. Laboratory data revealed normal disintegrin and metalloproteinase with a thrombospondin type 1 motif, member 13 (ADAMTS13) activity (check before the initiation of HD); hence, she was unlikely to have thrombotic thrombocytopenic purpura (TTP). Throughout the course, there was no obvious infection. Due to a complicated medical history and rapid course of AKI, it was difficult to perform a kidney biopsy and identify the definite cause of disease immediately after the onset of AKI. Imidapril, an angiotensin-converting enzyme inhibitor, 5 mg/day was initiated. Her urine volume gradually increased. She was able to withdraw from hemodialysis therapy two weeks after the initiation of imidapril.

To further examine the genetic involvement of the complement system, we performed extensive diagnostic testing to identify the cause of TMA. Samples for the extensive complement analysis were taken. Plasma-soluble C5b-9 complement levels were 526.4 (reference range: 37.0–260.6) ng/mL, complement factor Ba levels were 3,970.4 (275.6–685.2) ng/mL, CFH levels were 391.7 (229.8–714.6) *µ*g/mL, and complement 5a levels were 15.84 (0.20–15.62) ng/mL. Enzyme-linked immunosorbent assay for anti-CFH IgG was negative (510.6 (393.9–1069.0) AU/mL). After obtaining informed consent, we collected DNA from the patient. Genomic DNA was extracted from whole blood, and targeted next-generation sequencing of candidate genes for aHUS was performed. Sequencing of the exonic regions of 9 genes (CFH, *CFHR5*, *C3*, *CFI*, *CFB*, *MCP*, *THBD*, *DGKE*, and *PLG*) conducted at the Japanese Association for Complement Research revealed a heterogeneous very rare missense variant (exon 5; NM_000186.3:c.526 T > *C*: p.Phe176Leu) in the CFH gene. Despite the CFH variant, serum and plasma CFH levels were normal. It was not possible to completely deny the possibility that CFH p.Phe176Leu may have some influence as a causative genetic mutation; however, we did not use eculizumab (ECZ). A diagnosis of SLE was considered on account of reduced C3 and C4 counts along with the gradual effect of steroid treatment. However, the hemolytic anemia was attributable to TMA, and there were no other findings to suspect lupus nephritis; hence, a clear diagnosis of SLE could not be made.

The subsequent clinical course was relatively better. Hemodialysis was discontinued. Two years later, her creatinine level stayed at 1.91 mg/dl.

## 3. Discussion

We reported the case of a patient with the CFH gene variant (p.Phe176Leu in exon 5) who developed TMA on a mixed clinical background. Kidney biopsy findings confirmed that the patient had TMA; however, her medical history was complex. The etiology of TMA is complex, and in some cases, it is difficult to identify the cause. Bayer et al. [4] found that primary TMA accounted for only about 6% of the 564 patients in their study, while the remaining 94% patients had secondary TMA. In addition, they reported that the etiology in 57% of secondary TMA cases was multifactorial. As showed in the case presentation, it must be acknowledged that the diagnosis of background disease was inadequate; however, it was a characteristic finding that the TMA had a CFH variant. Although hypocomplementemia and renal dysfunction are pathological conditions indicating SLE, there were no findings of lupus nephritis, and a clear diagnosis of SLE could not be made.

In aHUS, mutation of CFH is identified in approximately 25% of sporadic cases and in 40% of familial cases [5]. CFH is the most frequent causative gene of aHUS in Europe and the United States (20–30%); however, in Japan, it is <10% [6]. Genetic variations in the CFH gene family are associated with several complex diseases, such as aHUS, C3 glomerulopathy, and age-related macular degeneration (AMD) [7]. In our case, a p.Phe176Leu variant of CFH was revealed. The p.Phe176Leu variant has been reported as a suspected pathological mutation of aHUS [6]. However, that reported case had another aHUS-predisposing variant (p.Arg1215Gln) with known pathogenicity [6]. On the other hand, p.Phe176Leu has recently been reported as a mutation capable of causing AMD [8]. AMD is a disease that is caused by complement dysregulation [9]; hence, it is undeniable that the p.Phe176Leu variant is involved in the amplification of complement activation by the alternative pathway. The p.Phe176Leu variant occurs in the cofactor region of CFH. In our patient, it could not be denied that it solely may lead to a reduction in cofactor activity and may be involved in the amplification of complement activation by the alternative pathway. p.Arg175Trp, an adjacent amino acid of p.Phe176Leu, was reported to be a potentially pathogenic mutation of aHUS [10]. Whether p.Phe176Leu is involved in the pathological condition cannot be determined without a functional analysis; therefore, further studies are required.

Currently, ECZ, a monoclonal antibody that targets complement protein C5, is widely used as a treatment for complement-related HUS [11]. To date, the pathogenesis of secondary HUS has not been fully studied; however, complement dysregulation is involved in some cases of secondary HUS [12]. In general, as shown in our case, the use of ECZ is not required for secondary TMA; however, the use of ECZ for secondary TMA is controversial. Even in some cases of secondary TMA, ECZ is very effective for rapidly resolving TMA and improving renal function [12]. In patients with refractory secondary SLE- and/or antiphospholipid syndrome-related TMA, despite treatment of lupus nephritis, the use of ECZ in 9 patients showed a positive outcome in hematology values, renal function, and other organ functions following treatment with ECZ, suggesting that it involves complement pathways. Unfortunately, a genetic analysis of complement genes was not performed in that study [13]. ECZ is a very effective drug for aHUS; however, ECZ is a very expensive drug. The kind of patient that should be treated and how long treatment should continue are considered important issues. The usefulness of ECZ for secondary TMA should be further investigated in future studies.

We reported the case of a patient with the CFH variant who developed TMA on a mixed clinical background. The pathophysiology of secondary TMA is complex, and it is important to keep in mind that the presence of complement gene abnormalities in secondary TMA should be investigated to help determine whether ECZ should be used.

## Figures and Tables

**Table 1:** Laboratory data upon the first admission of the patient.

Examination	Value	Reference range	Examination	Value	Reference range	Examination	Value	Reference range
(Hematology)			(Coagulation)			(Serology; continue)		
WBC (/*µ*L)	6690	3300–8600	aPTT (second)	23.9	26.9–40.9	ADAMTS13	72.0	70–120
RBC (10^4^/*µ*L)	349	386–492	PT (%)	117	70–130	IL-2R (U/ml)	1214	122–496
Hb (g/dL)	9.9	11.6–14.8	Fibrinogen (mg/dl)	438	200–400	Cryoglobulin	—	
Ht (%)	30.5	35.1-44-4	FDP (*µ*g/ml)	25.5	<5	CEA (ng/ml)	2.3	<5.8
Plt (× 10^3^/*µ*L)	17.8	15.8–34.8	D-dimer (*µ*g/ml)	7.2	<1.0	CA19-9 (U/ml)	11	<37
						CA125 (U/ml)	112	<35
(Biochemistry)			(Serology)					
Total protein (g/dl)	6.4	6.6–8.1	CRP (mg/dl)	2.47	<0.14	(Infectious marker)		
Albumin (g/dl)	2.8	4.1–5.1	IgG (mg/dl)	1008	861–1747	HBs Ag	—	
Total bilirubin (mg/dl)	0.6	0.4–1.5	IgA (mg/dl)	97	93–393	HBs ab	—	
ALT (U/L)	30	13–30	IgM (mg/dl)	206	50–269	HCV ab	—	
AST (U/L)	11	7–23	C3 (mg/dl)	41.8	73–138	HIV ab	—	
LDH (U/L)	485	124–222	C4 (mg/dl)	<5	11–31	TPHA	—	
ALP (U/L)	427	106–322	CH50 (U/ml)	14	30–46	STS	—	
*γ*GTP (U/L)	94	9–32	IC-1q (*µ*g/ml)	<1.5	<3.0			
BUN (mg/dl)	36	8–20	ANA (INDEX)	72.4	<20	(Urinalysis)		
Cre (mg/dl)	2.55	0.46–0.79	Anti-DNA (RIA)	2.3	<6	Protein	3+	
UA (mg/dl)	7.5	2.6–5.5	Anti-SS-A (INDEX)	149.0	<10	Protein (g/gCr)	4.7	
Na (mEq/l)	140	138–145	Anti-SS-B (INDEX)	<5.0	<15	Glucose	—	
K (mEq/l)	3.1	3.6–4.8	Anti-SM (INDEX)	<5.0	<7	Ketone	—	
Cl (mEq/l)	105	101–108	Anti-Scl-70 (INDEX)	<5.0	<16	Occult blood	3+	
Ca (mg/dl)	7.9	8.8–10.1	Anti-CENPB (INDEX)	<5.0	<10	Sediments		
iP (mg/dl)	3.7	2.7–4.6	Anti-U1 RNP (INDEX)	<5.0	<15	Red blood cells (/hpf)	50–99	
Glucose (mg/dl)	149	73–109	Anti-ARS (INDEX)	<5.0	<25	White blood cells (/hpf)	5–9	
HbA1c (%)	4.8	4.9–6.0	Anti-CLGPI (U/mL)	<1.2	<3.5	Hyaline casts (/hpf)	10–99	
HDL-C (mg/dl)	61	48–103	aPL IgG (U/ml)	<8	<10	B2MG (*µ*g/L)	158	13.0–301.2
LDL-C (mg/dl)	113	65–163	Anti-CCP (U/ml)	<0.5	<4.5			
Triglyceride (mg/dl)	68	30–117	MPO-ANCA (IU/mL)	<1.0	<3.5	(Blood gas analysis)		
			PR3-ANCA (IU/mL)	<1.0	<2.0	pH	7.477	7.35–7.45
			Anti-GBM (IU/mL)	2.0	<3	pCO2 (mmHg)	31.9	32–45
			BNP (pg/dl)	1055	<18.4	pO2 (mmHg)	77.0	83–108
			Ferritin (ng/dl)	210	5–152	HCO3^−^ (mmol/L)	21.5	21–28
			Haptoglobin (mg/dl)	<10	19–170	Base excess (mmol/L)	−2.2	

WBC: white blood cell, RBC: red blood cell, Hb: hemoglobin, Ht: hematocrit, Plt: platelet, ALT: alanine aminotransferase, AST: aspartate aminotransferase, LDH: lactate dehydrogenase, ALP: alkaline phosphatase, *γ*-GTP: *γ*-glutamyltranspeptidase, BUN: blood urea nitrogen, Cre: creatinine, UA: uric acid, Na: sodium, K: potassium, Cl: chlorine, Ca: calcium, iP: inorganic phosphorus, HbA1c: hemoglobin A1c, HDL-C: high-density lipoprotein cholesterol, LDL-C: low-density lipoprotein cholesterol, aPTT: activated partial thromboplastin time, PT: prothrombin time, FDP: fibrin/fibrinogen degradation products, CRP: C-reactive protein, IgG: immunoglobulin G, IgA: immunoglobulin A, IgM: immunoglobulin M, C3: complement 3, C4: complement 4, CH50: total complement immunoassay, IC-C1q: immune complex C1Q binding assay, ANA: antinuclear antibody, Anti-DNA: anti-DNA antibody, Anti-SS-A: anti-SS-A antibody, Anti-SS-B: anti-SS-B antibody, Anti-SM: anti-Smith antibody, Anti-Scl-70: antitopoisomerase antibody, Anti-CENPB: anticentromere autoantigen B antibody, Anti-U1 RNP: antiribonucleoprotein antibody, Anti-ARS: antiaminoacyl tRNA synthetase antibody, Anti-CLGPI: anticardiolipin antibody, aPL: antiphospholipid antibody, Anti-CCP: anticyclic citrullinated peptide antibody, MPO-ANCA: myeloperoxidase-antineutrophil cytoplasmic antibody, PR3-ANCA: proteinase-3-antineutrophil cytoplasmic antibody, Anti-GBM: antiglomerular basement membrane antibody, BNP: brain natriuretic peptide, ADAMTS13: a disintegrin and metalloproteinase with a thrombospondin type 1 motif, member 13, IL-2R: soluble interleukin-2 receptor, CEA: carcinoembryonic antigen, CA19-9: carbohydrate antigen 19–9, CA125: cancer antigen 125, HBs Ag: hepatitis B surface antigen, HBs Ab: hepatitis B surface antibody, HCV Ab: hepatitis C antibody, HIV Ab: human immunodeficiency virus antibody, TPHA: Treponema pallidum hemagglutination assay, STS: serologic test for syphilis, hpf: high-power field, B2MG *β*2: microglobulin, pCO2: carbon dioxide partial pressure, pO2: oxygen partial pressure, HCO3^−^: bicarbonate ion.

## Data Availability

The data that support the findings of this study are available from the corresponding author, Y.I., upon reasonable request.
